# We’re in This Together, but . . . : Community-Engaged Research Process of an Arts Intervention With Older Adults

**DOI:** 10.1093/geroni/igaa057.2966

**Published:** 2020-12-16

**Authors:** Sudha Sudha, Lia Miller

**Affiliations:** 1 UNC Greensboro, Greensboro, North Carolina, United States; 2 Creative Aging Network NC, Greensboro, North Carolina, United States

## Abstract

Community engaged (CE) methods are used in health-related research, but few discuss methodological aspects among older adults. We describe the methodology and lessons learned from a CE study of whether ARTmail, a structured participatory arts program, benefited older adults aged 60+ with memory symptoms / cognitive impairment (MS/CI). Our study, conducted in 2015-2017 with support from the NEA, was a partnership between a non-profit organization in North Carolina that provides creative programming for older adults with varied abilities, and researchers in an area University. Older adults with MS/CI symptoms receiving care in area communities were recruited into either the art intervention or a control group, in collaboration with community staff. We reflect on the CE research process with a partnership among community organizations, researchers, care staff, and older adults. Asymmetries in priorities, resources, and decision-making power are described, and implications for the research process and findings are discussed.

